# Active pediatric HIV case finding in Kenya and Uganda: A look at missed opportunities along the prevention of mother-to-child transmission of HIV (PMTCT) cascade

**DOI:** 10.1371/journal.pone.0233590

**Published:** 2020-06-02

**Authors:** Michelle M. Gill, Eliab K. Natumanya, Heather J. Hoffman, Gordon Okomo, Geoffrey Taasi, Laura Guay, Rose Masaba

**Affiliations:** 1 Elizabeth Glaser Pediatric AIDS Foundation, Washington, DC, United States of America; 2 Elizabeth Glaser Pediatric AIDS Foundation, Mbarara, Uganda; 3 Department of Biostatistics and Bioinformatics, Milken Institute School of Public Health, George Washington University, Washington, DC, United States of America; 4 Kenya Ministry of Health, Homa Bay County, Nairobi, Kenya; 5 Uganda Ministry of Health, Kampala, Uganda; 6 Elizabeth Glaser Pediatric AIDS Foundation, Nairobi, Kenya; AIDS Healthcare Foundation, UNITED STATES

## Abstract

**Background:**

Children living with HIV remain undiagnosed due to missed opportunities along the prevention of mother-to-child HIV transmission cascade. This study addresses programmatic gaps in the cascade by describing pregnancy and HIV-related services received by mothers of children newly identified as HIV-positive through active case finding.

**Methods:**

This was a prospective observational cohort (2017–2018) of HIV-positive children <15 years of age newly diagnosed at study facilities and/or surrounding communities in Kenya and Uganda. At enrollment, caregivers were interviewed about maternal and child health and HIV history. Child medical and laboratory information was abstracted at two months post-diagnosis. Descriptive summary statistics were calculated; associations between selected factors and child age at HIV diagnosis were evaluated using generalized estimating equations.

**Results:**

174 HIV-positive children (median age 2.4 years) were enrolled. Among maternal caregivers, 110/132 (83.3%) attended antenatal care and 60 (45.5%) reported testing HIV-negative in antenatal care. Of 41 and 56 women known to be HIV-positive during pregnancy and breastfeeding respectively, 17 (41.5%) and 15 (26.8%) did not receive antiretroviral drugs. Despite known maternal HIV-positive status during pregnancy, 39% of these children were not diagnosed until after two years of age; children were diagnosed at younger ages in Uganda (p = 0.0074) and if mother was the caregiver (p<0.0001). The most common HIV testing points identifying children were outpatient (44.3%) and maternal/child health departments (29.9%). Nearly all children initiated antiretroviral therapy within two weeks of diagnosis.

**Conclusions:**

Multiple missed opportunities for HIV prevention and delays in HIV testing of HIV-exposed children were identified in newly diagnosed children. Findings support critical prevention messaging and retesting of HIV-negative women during pregnancy and breastfeeding, strengthening HIV treatment initiation and follow-up systems and interventions to ensure HIV-positive women receive lifelong antiretroviral therapy throughout the cascade, and broader implementation of community case finding so children not engaged in care receive testing services.

## Introduction

Accelerated progress in preventing new pediatric HIV infections and treating HIV-positive infants is imperative for decreasing the morbidity and mortality of children worldwide. Massive scale-up of prevention of mother-to-child HIV transmission (PMTCT) services, HIV testing services (HTS), antiretroviral therapy (ART), and HIV prevention services have resulted in a 35% decrease in new pediatric infections between 2010 and 2017 [1,2]. Yet, in 2017, an estimated 180,000 children ages 0–14 years were newly infected with HIV. Of the 1.8 million children living with HIV, only 52% had access to ART [1]. Early identification of HIV infection in children followed by rapid ART initiation is critical to their survival [3]. HIV-exposed children should receive HIV DNA-PCR testing within two months of birth and be provided prompt treatment if diagnosed as HIV-infected. However, only half of HIV-exposed infants receive early infant diagnostic testing by eight weeks of age and far fewer are retested following breastfeeding cessation [4]. Intensified efforts are needed to reach UNAIDS’ “95-95-95” global targets of 95% of people living with HIV (PLHIV) identified, 95% of those infected on ART and 95% of those on ART with viral suppression by 2030, especially among children.

Mother-to-child transmission continues to account for over 90% of new pediatric infections, yet significant numbers of children remain undiagnosed. Adhering to all steps in the PMTCT cascade, which begins with all pregnant women and ends with either a final HIV-negative test result after breastfeeding cessation for HIV-exposed infants or a diagnosis of HIV infection [5], is needed to prevent vertical transmission and diagnose new infections early. These steps include testing pregnant women and their partners of unknown HIV status early in antenatal care (ANC), initiating HIV-positive women on ART, retaining women in care through delivery and postpartum, retesting HIV-negative women, encouraging recommended breastfeeding practices, and providing early infant diagnosis and final HIV antibody testing after breastfeeding. Focusing on reaching all pregnant mothers and children throughout the PMTCT cascade will decrease the number of new pediatric infections and link HIV-positive children to care and treatment.

There are numerous facility and community testing venues and testing strategies used to identify HIV-positive infants, yet barriers to pediatric HIV testing remain. Strategies include provider-initiated testing and counseling (PITC), voluntary counseling and testing (VCT) services, testing campaigns and index testing, a process in which providers elicit a list of sexual (or injectable drug-using) partners and children of index clients, the individuals first identified as HIV-positive, and offer HIV testing to those contacts [6–10]. Index testing in which contacts are identified and tested at home provides a promising opportunity to reach children [9,11,12]. Barriers that inhibit the success of testing strategies include the distance from testing facilities, long wait times, shortages of test kits or laboratory reagents, fear of stigma and concerns about privacy, opposition from partners and family, and loss to follow-up along the PMTCT cascade [6,13]. Like most health interventions, the success of HIV testing strategies is context-dependent, thus considering the challenges and characteristics of unique settings is necessary to determine the feasibility and effectiveness of different models for early identification of pediatric HIV infections.

This study aimed to describe the PMTCT services received by the mothers of children newly identified as HIV-positive in selected facilities and surrounding communities in Kenya and Uganda. An understanding of what steps in the PMTCT cascade were missed can target efforts to strengthen these services and prevent future pediatric infections. We also described children’s testing entry points and the strategies through which they were identified as HIV-positive as well as their HIV testing history and clinical profiles at diagnosis to inform approaches to pediatric case finding, including for older children who may have limited interactions with the health system.

## Materials and methods

### Setting

This study was conducted in Homa Bay County, Kenya and the southwest region of Uganda. Homa Bay is a county in western Kenya with high HIV prevalence in adults (20.7%), while southwest Uganda is a region of medium HIV prevalence in adults (7.9%) [14,15]. In Kenya and Uganda respectively, 110,000 and 95,000 children aged 0 to 14 years were estimated to live with HIV in 2017 [16,17].

In both countries, several strategies for identifying HIV-positive children < 15 years of age are used by Ministry of Health (MOH) programs, with support of the Elizabeth Glaser Pediatric AIDS Foundation (EGPAF). As opposed to passive case identification, active case finding requires additional targeted efforts to identify affected individuals. Active case finding strategies include testing children attending all outpatient and inpatient services and in key service delivery areas (e.g., malnutrition, tuberculosis), index testing, door-to-door testing, and community testing campaigns. Kenya provides ART for children identified as HIV-positive, with universal “Test and Start” guidelines implemented in July 2016 [18]. A “Test and Start” policy for HIV-positive children <15 years of age has been in place in Uganda since 2014 [19].

### Study design, population and sampling

This was a prospective observational cohort study of HIV-positive children <15 years of age who were newly diagnosed at either a study facility or surrounding study community where HIV testing was offered and their mothers or primary caregivers. Children were enrolled at the time of HIV diagnosis with follow up for two months.

Children were enrolled from 45 selected facilities: 15 facilities in six sub-counties of Homa Bay and 30 facilities in 12 districts of southwest Uganda. Sites were purposively selected to include low- (1–9 HIV-positive children identified in six months) and high- (≥ 10 HIV-positive children identified in six months) volume facilities with a mix of sub-county hospitals, health centers, and dispensaries in Kenya and hospitals and health centers IV, III and II in Uganda. In total, there were 25 low-volume and 20 high-volume study facilities.

Health facility staff working in various clinics and departments referred caregivers of newly diagnosed potentially eligible children to study staff based at study facilities. Health workers at study facilities also conducted community outreach and were accompanied by study staff to the extent possible. In Kenya, children testing HIV-positive through community-based testing programs were referred to health facilities for confirmation of HIV status and linkage to HIV care and treatment services, where they were then screened for study eligibility. In Uganda, participants were primarily enrolled in the communities.

Prior to the initiation of study procedures, written informed consent was obtained from mothers or caregivers of newly diagnosed HIV-positive children by trained study staff. In Uganda, non-emancipated minors 8–14 years of age were asked to provide written assent following their caregivers’ consent, while in Kenya, a waiver of assent from children was approved by the Institutional Review Board (IRB).

### Data collection and statistical analysis

At enrollment, caregivers were interviewed to collect caregiver and child demographics and caregiver HIV and ART status (whether or not the caregiver was the child’s mother). If the biological mother or father was the caregiver interviewed, they were also asked about mother’s history of ANC attendance and HIV testing and ARV/PMTCT drug use in ANC if HIV-positive. All caregivers were also asked about the child’s previous HIV testing and testing entry point for the current HIV test. Enrollment took place November 2017 to May 2018 in Kenya and March to October 2018 in Uganda. Study staff abstracted child medical and laboratory information from clinic registers at two months post-diagnosis, including child ART initiation date, clinical conditions, and any follow-up visits. If records were incomplete, study staff contacted the caregiver or visited the household in a few cases (Uganda only) to obtain outcome information (linked to care at another facility, defaulted from appointments, or deceased).

Descriptive summary statistics were calculated for outcome variables of interest using frequencies and percentages for categorical variables, and medians and interquartile ranges and mean and standard deviations for continuous variables. Associations between sociodemographic, clinical and facility characteristics and child age at HIV diagnosis were evaluated using unadjusted and adjusted generalized estimating equations (GEE) having a normal distribution with an identity link function and a compound symmetry working correlation structure. Analyses included both country-specific and combined country modeling. Only those variables significant at the 0.20 level in the unadjusted GEE models were considered for the adjusted GEE model. All statistical tests were two-sided and the level of statistical significance was set at 0.05.

### Ethical approval

The protocol was approved by the George Washington University IRB in the United States, the African Medical Research Foundation’s Ethical and Scientific Research Committee in Kenya, and the National AIDS Research Committee in Uganda.

## Results

**Fig 1:**
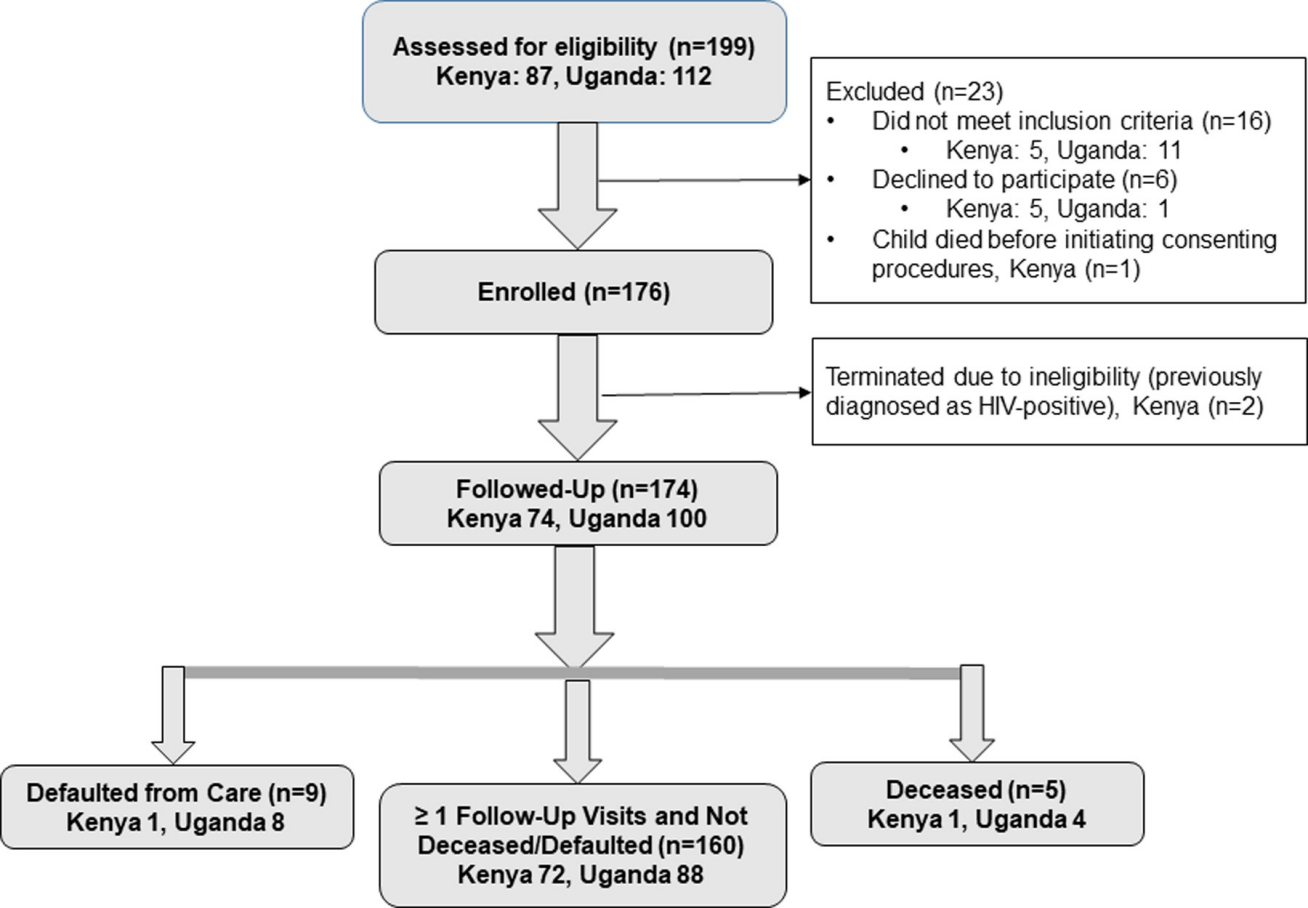
Screening, enrollment and follow-up.

In total, 176 children and their caregivers were enrolled in the study; however, two children were excluded in Kenya due to ineligibility (final Kenya: 74, Uganda: 100) (Fig 1).

The overall median age of caregivers in Kenya and Uganda was 29 years (interquartile range [IQR] 24–38) (Table 1). The median age of children was 2.4 years (IQR 0.9–6.2). Children were slightly older in Kenya (3.6 years) than in Uganda (2.0 years). The majority (69.5%) of caregivers were the child’s biological mother. Overall, 138 (79.3%) of the caregivers (98.3% of mothers) were HIV-positive and 86.2% of HIV-positive caregivers were on ART.

**Table 1:** Child, caregiver and household information.

	Kenya N = 74	Uganda N = 100	TOTAL N = 174
**Children**			
Child age, Median (IQR)	3.6 (1.4–8.8)	2.0 (0.9–4.5)	2.4 (0.9–6.2)
**Child age**	**N (%)**	**N (%)**	**N (%)**
<2 years	23 (31.1)	50 (50.0)	73 (42.0)
2–9 years	37 (50.0)	45 (45.0)	82 (47.1)
> 9 years	14 (18.9)	5 (5.0)	19 (10.9)
**Child sex**			
Female	36 (48.6)	54 (54.0)	90 (51.7)
**Caregivers**			
Caregiver age, Median (IQR)	30.0 (24.0–38.0)	29.0 (24.0–38.5)	29.0 (24.0–38.0)
Biological mother age (n = 121), Median (IQR)	27.0 (24.0–31.0)	25.0 (21.0–31.0)	26.0 (22.0–31.0)
**Biological mother age**	**N (%)**	**N (%)**	**N (%)**
<19 years	1 (1.9)	6 (8.8)	7 (5.8)
19–24 years	18 (34.0)	23 (33.8)	41 (33.9)
>24 years	34 (64.2)	39 (57.4)	73 (60.3)
**Caregiver sex**			
Female	64 (86.5)	90 (90.0)	154 (88.5)
**Caregiver’s relationship to child**			
Biological mother	53 (71.6)	68 (68.0)	121 (69.5)
Biological father	7 (9.5)	6 (6.0)	13 (7.5)
Grandparent	3 (4.1)	15 (15.0)	18 (10.3)
Other relative	11 (14.9)	11 (11.0)	22 (12.6)
**Caregiver’s marital status**			
Married or living with partner as a couple	56 (75.7)	51 (51.0)	107 (61.5)
Never married or not living with partner	3 (4.1)	6 (6.0)	9 (5.2)
Divorced/separated	6 (8.1)	30 (30.0)	36 (20.7)
Widowed	9 (12.2)	13 (13.0)	22 (12.6)
**Caregiver education level**			
Never attended school	4 (5.4)	13 (13.0)	17 (9.8)
Primary	48 (64.9)	64 (64.0)	112 (64.4)
Secondary/ Post-secondary	16 (21.6)	16 (16.0)	32 (18.4)
Tertiary	6 (8.1)	7 (7.0)	13 (7.5)
**Caregiver HIV status**			
HIV-positive	62 (83.8)	76 (76.0)	138 (79.3)
**Caregiver currently on ART**	**n = 62**	**n = 76**	**n = 138**
Yes	56 (90.3)	63 (82.9)	119 (86.2)

### Antenatal and postpartum care

Information on mothers’ antenatal care is presented only from biological mother and father caregivers. Information from two biological mothers is excluded from Table 2 below, as they reported not being HIV-positive, thus ruling out mother-to-child transmission (MTCT) in their children. One child in Kenya (14 years of age) was presumed to be infected through sexual transmission; another child in Uganda (8 years of age) was reportedly raped by a relative. Data from 132 biological caregivers are reported in Table 2 (Kenya: 59, Uganda: 73). In Kenya, 47 (79.7%) and in Uganda, 63 (86.3%) mothers attended ANC; two known HIV-positive women in each country did not attend ANC. In total, of the 93 mothers who had an HIV test in ANC, 60 (64.5%) initially tested HIV-negative leading to 45.5% overall who reported testing HIV-negative in ANC. Of 41 and 56 known HIV-positive women during pregnancy and breastfeeding respectively, 17 (41.5%) and 15 (26.8%) did not receive antiretroviral (ARV) drugs. Reasons included clinic not offering ARVs as part of antenatal care or during breastfeeding, defaulting from clinic, home delivery, HIV status denial, and other/unknown reasons (e.g., financial, religious). Overall 36.6% of mothers did not deliver in a facility. Of those with breastfeeding information available, mothers breastfed their children for a median 13 months (IQR 6–24) in Kenya (n = 62) and 12 months (IQR 6–18) in Uganda (n = 82), though some mothers were still breastfeeding at the time of the interview; eight caregivers in Uganda reported no breastfeeding.

**Table 2:** Maternal and child health and HIV health-seeking behavior^+^.

	Kenya	Uganda	TOTAL
	**n = 59 N (%)**	**n = 73 N (%)**	**n = 132 N (%)**
**Mother attended ANC**			
Yes	47 (79.7)	63 (86.3)	110 (83.3)
No	10 (17.0)	8 (11.0)	18 (13.6)
Unknown	2 (3.4)	2 (2.7)	4 (3.0)
**Mother received HIV test in ANC**	**n = 47**	**n = 63**	**n = 110**
Yes	40 (85.1)	53 (84.1)	93 (84.5)
No-known HIV-positive*	4 (8.5)	3 (4.8)	7 (6.4)
No-not offered	1 (2.1)	7 (11.1)	8 (7.3)
Unknown	2 (4.3)	0	2 (1.8)
**Mother’s HIV test result in ANC**	**n = 40**	**n = 53**	**n = 93**
HIV-positive	14 (35.0)	16 (30.2)	30 (32.3)
HIV-negative	24 (60.0)	36 (67.9)	60 (64.5)
Unknown	2 (5.0)	1 (1.9)	3 (3.2)
**Mother received ARVs during ANC**	**n = 20**	**n = 21**	**n = 41**
Yes–ARV prophylaxis	10 (50.0)	5 (23.8)	15 (36.6)
Yes–ART to be taken for life	3 (15.0)	6 (28.6)	9 (22.0)
No	7 (35.0)	10 (47.6)	17 (41.5)
**Of those who did not receive ARVs, why not?**	**n = 7**	**n = 10**	**n = 17**
Never offered by health facility	3	4	7
Did not attend clinic^	2	1	3
Other	2	5	7
**Mother received ARVs during delivery/breastfeeding**	**n = 21**	**n = 35**	**n = 56**
Yes–ARV prophylaxis	12 (57.1)	6 (17.1)	18 (32.1)
Yes–ART to be taken for life	2 (9.5)	21 (60.0)	23 (41.1)
No	7 (33.3)	8 (22.9)	15 (26.8)
**Of those who did not receive ARVs, why not?**	**n = 7**	**n = 8**	**n = 15**
Never offered by health facility	2	3	5
Delivered child at home	0	3	3
Mother refused to accept HIV+ status (in denial)	2	0	2
Stopped taking her drugs	2	0	2
Other	1	2	3
**Facility delivery of child**	**n = 73**	**n = 99**	**n = 172**
Yes	39 (53.4)	59 (59.6)	98 (57.0)
No	28 (38.4)	35 (35.4)	63 (36.6)
Unknown	6 (8.2)	5 (5.1)	11 (6.4)

+With the exception of facility delivery, only the responses of the biological mother or father are reported in this section.

*In Kenya, there were six mothers in total who were known to be HIV-positive prior to the pregnancy, but two did not attend ANC. In Uganda, there were five mothers in total who were known to be HIV-positive prior to the pregnancy, but two did not attend ANC.

^One woman in Uganda who did not attend ANC cited religious reasons for not receiving ARVs during ANC.

### Child HIV testing

**Fig 2:**
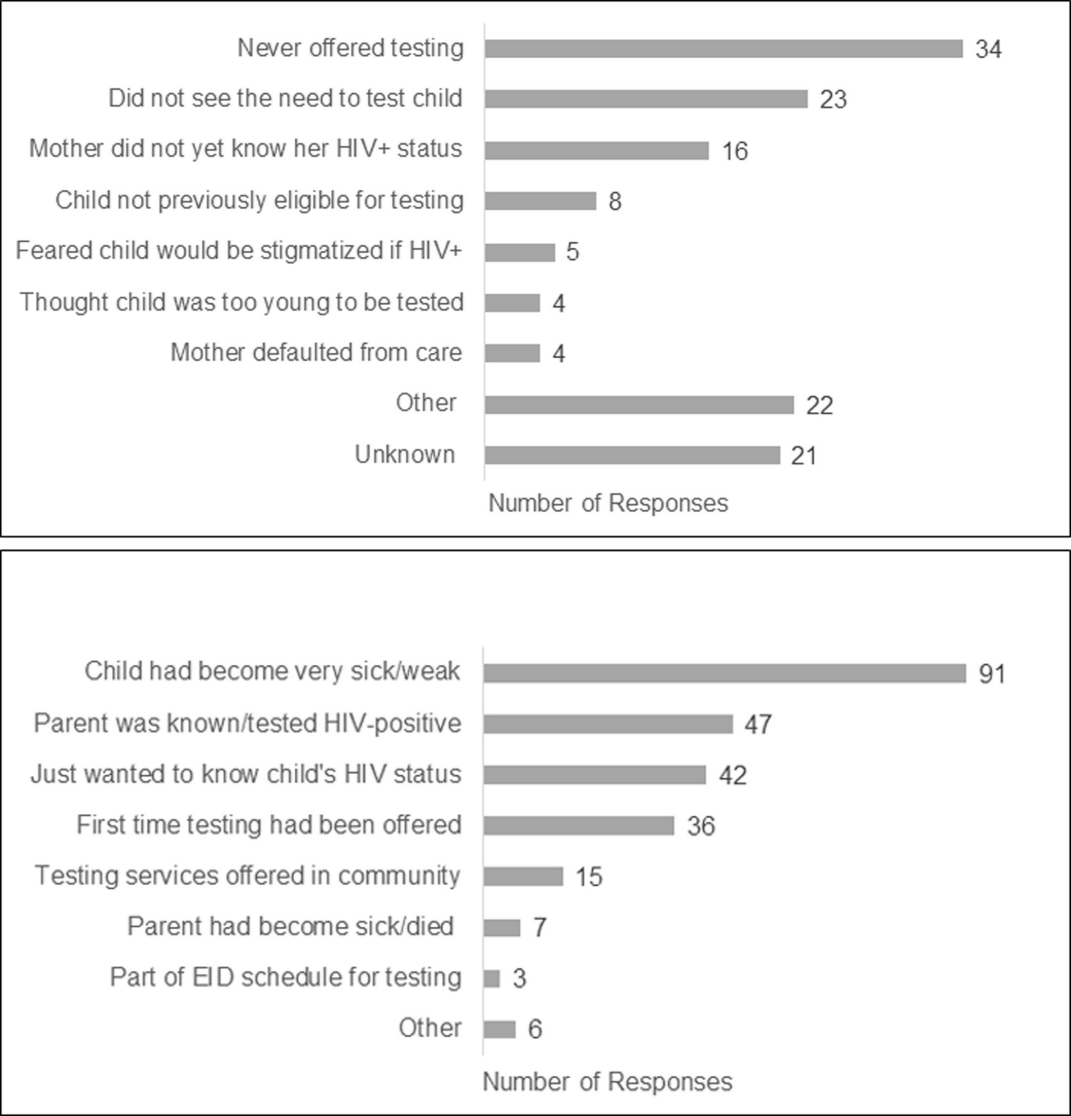
A. Reasons child was not tested previously for HIV. In Uganda only, some caregivers provided multiple responses for a total of 137 responses (n = 120). B. Reasons why child was tested today for HIV In both countries, some caregivers provided multiple responses for a total of 247 responses (n = 173).

Overall, 31 (17.8%) children were previously tested for HIV (Table 3). The two children infected through horizontal transmission had not received a prior HIV test. The majority of children (58.1%) had their last test at less than two years of age. In Kenya, all 17 children with a prior HIV test received an HIV-negative test result. In Uganda, eight received a negative result, three an indeterminate result, two never received the result, and one caregiver did not know the result; of the 25 children with a previous HIV- negative result, 16 were ≤ 2 years of age. The most common reasons for not testing was that testing was not offered to the child (n = 34), the caregiver did not see a reason to test the child or perceived the child to be healthy (n = 23), and that the mother did not yet know her HIV-positive status (n = 16) (Fig 2A). Of note, the same caregivers did not give more than one of these three common responses, except for two respondents who indicated that they did not see a reason to test the child and that the mother did not yet know her status. Other reasons cited for not testing the child were that the caregiver had not bothered or thought to do it and that the caregiver believed the child was ill for other reasons. About half of the unknown responses were from the biological mothers. The most common reason for testing the child on the day s/he was diagnosed as HIV-positive was that the child had become sick or weak (Fig 2B).

**Table 3:** Child HIV testing history and HIV-positive diagnosis.

	Kenya	Uganda	TOTAL
	**N = 74 N (%)**	**N = 100 N (%)**	**N = 174 N (%)**
**Child tested previously for HIV**			
Yes	17 (23.0)	14 (14.0)	31 (17.8)
No	50 (67.6)	80 (80.0)	130 (74.7)
Unknown	7 (9.5)	6 (6.0)	13 (7.5)
**Age most recent HIV test performed**	**n = 17**	**n = 14**	**n = 31**
< 24 months	9 (52.9)	9 (64.3)	18 (58.1)
2–4 years	2 (11.8)	0	2 (6.5)
≥ 5 years and above	5 (29.4)	5 (35.7)	10 (32.3)
Unknown	1 (5.9)	0	1 (3.2)
**Most recent HIV test result**	**n = 17**	**n = 14**	**n = 31**
HIV-negative	17 (100.0)	8 (57.1)	25 (80.6)
Indeterminate	0	3 (21.4)	3 (9.7)
Never received the result	0	2 (14.3)	2 (6.5)
Unknown	0	1 (7.1)	1 (3.2)
**Testing entry point (on day of HIV-positive test)**	**N = 74**	**N = 100**	**N = 174**
Outpatient department (OPD)	33 (44.6)	44 (44.0)	77 (44.3)
Maternal and child health (MCH)/PMTCT	21 (28.4)	31 (31.0)	52 (29.9)
Inpatient department (IPD)	4 (5.4)	11 (11.0)	15 (8.6)
Community outreach/child health days	7 (9.5)	8 (8.0)	15 (8.6)
Home-based/door-to-door testing	4 (5.4)	4 (4.0)	8 (4.6)
HIV clinic (comprehensive care clinic in Kenya)	2 (2.7)	0	2 (1.1)
Under 5 clinic	0	2 (2.0)	2 (1.1)
OVC service organization	2 (2.7)	0	2 (1.1)
Voluntary medical male circumcision	1 (1.4)	0	1 (0.6)
**Testing approach (on day of HIV-positive test)**	**N = 74**	**N = 100**	**N = 174**
PITC	49 (66.2)	68 (68.0)	117 (67.2)
VCT	13 (17.6)	22 (22.0)	35 (20.1)
Index	6 (8.1)	10 (10.0)	16 (9.2)
Other	6 (8.1)	0	6 (3.4)

Most enrolled HIV-positive children were identified in the outpatient department (OPD) (44.3%) and MCH/ PMTCT entry points (29.9%), followed by inpatient department (IPD) and community outreach (Table 3). Trends were similar for the individual countries, except a greater proportion of HIV-positive children were identified in IPD in Uganda (11% vs. 5% in Kenya). Most of the newly diagnosed children enrolled in the study were identified through PITC.

### Timing of maternal and child HIV diagnoses

Of the 41 mothers who were known or tested HIV-positive during pregnancy, 25 (61.0%) of their infants were diagnosed by age two years, while eight children (19.5%) were >2 - <5 years when diagnosed, and eight were not diagnosed until age 5–12 years, though the caregivers of seven children >2–12 years reported their child previously tested HIV-negative (Kenya only). Of 76 mothers with HIV diagnosis after delivery and with complete mother-child HIV diagnosis information, 26 (34.2%) mothers and children were diagnosed on the same day and 31 (40.8%) of their children were diagnosed as HIV-positive between one day and two months after the mother tested HIV-positive. Eight children (10.5%) were not diagnosed until 1–7 years after the mother tested HIV-positive, two of whom reportedly tested HIV-negative prior to the current test. In Kenya, an additional three children were diagnosed before maternal diagnosis of HIV.

In adjusted GEE models, children were diagnosed at younger ages if the mother/child pair was in Uganda (p = 0.0074) and if the biological mother was the caregiver (p<0.0001) (Table 4). Caregiver’s educational background, caregiver’s ART status, the mother’s timing of HIV diagnosis (pregnancy versus after delivery), presence of child symptoms/opportunistic infections (OI), and the health facility location and level were not found to be statistically significantly associated with child age at HIV diagnosis in the combined country analyses. Modeling for each country individually found the caregiver relationship significantly associated (Kenya: p<0.0001, Uganda: p = 0.0040). In Uganda only, children were diagnosed at a younger age in urban compared to rural facilities (p = 0.030); higher-level facilities (e.g., hospitals) were marginally significantly associated with younger age at diagnosis (p = 0.044).

**Table 4:** Mean child age at diagnosis estimated from unadjusted and adjusted GEE models.

Variable	Unadjusted			Adjusted		
	Mean	95% CI	p-value	Mean	95% CI	p-value
**Country**			**0.0041**			**0.0074**
Kenya	5.01	(3.84, 6.17)		5.73	(4.71, 6.76)	
Uganda	2.97	(2.22, 3.73)		3.84	(2.87, 4.81)	
**Caregiver’s education status**			0.79			N/A
None	3.30	(1.67, 4.93)				
Some or all primary	3.86	(3.09, 4.63)				
Secondary or more	3.51	(2.28, 4.73)				
**Caregiver's relationship to child**			**<0.0001**			**<0.0001**
Mother	2.76	(2.12, 3.40)		3.12	(2.42, 3.83)	
Other	6.01	(4.75, 7.26)		6.45	(5.28, 7.62)	
**Caregiver currently on ART**			0.68			N/A
Yes	3.37	(2.67, 4.08)				
No	3.10	(1.72, 4.47)				
**Mother diagnosed as HIV+ after delivery**			0.21			N/A
No	2.45	(1.49, 3.42)				
Yes	3.03	(2.34, 3.71)				
**Presence child symptoms/OIs**			**0.18**			0.38
No	3.39	(2.56, 4.23)		4.60	(3.68, 5.53)	
Yes	4.09	(3.17, 5.01)		4.97	(4.25, 5.69)	
**Health facility location**			0.94			N/A
Rural	3.70	(2.78, 4.62)				
Urban	3.76	(2.68, 4.84)				
**Health facility level**			**0.06**			0.39
Low (dispensaries, health centers)	3.27	(2.44, 4.10)		4.46	(3.58, 5.34)	
High (hospitals)	4.69	(3.46, 5.91)		5.11	(3.93, 6.30)	

### Child clinical outcomes and linkage to care and treatment

At the time of enrollment into HIV care and treatment, nearly half of the children (45.9%) presented with one or more clinical symptoms, though the majority of children (88.3%) were assessed as WHO stage I or II (Table 5). Fewer children in Kenya than Uganda had clinical symptoms (38.9% vs. 51.0% respectively). The most common conditions were chronic upper respiratory tract infections, malnutrition, and weight loss, though the latter two were more common in Ugandan children.

**Table 5:** Child clinical profiles and linkage to HIV treatment.

	Kenya	Uganda	TOTAL
**Child conditions at time of HIV enrollment in care**	**n = 72 N (%)**	**n = 98 N (%)**	**n = 170 N (%)**
Present	28 (38.9)	50 (51.0)	78 (45.9)
Types of conditions*	**n = 28 (45 responses)**	**n = 50 (74 responses)**	**n = 78 (119 responses)**
Chronic upper respiratory tract infections	10 (22.2)	18 (24.3)	28 (23.5)
Malnutrition	5 (11.1)	16 (21.6)	21 (17.6)
Weight loss	5 (11.1)	15 (20.3)	20 (16.8)
Fever	5 (11.1)	7 (9.5)	12 (10.1)
Thrush	2 (4.4)	9 (12.2)	11 (9.2)
Tuberculosis	3 (6.7)	5 (6.8)	8 (6.7)
Skin rash	7 (15.6)	2 (2.7)	9 (7.6)
Failure to thrive	5 (11.1)	0	5 (4.2)
Other	3 (6.7)	2 (2.7)	5 (4.2)
**Child WHO stage at time of enrollment in care**	**n = 71**	**n = 100**	**n = 171**
Stage I	44 (62.0)	57 (57.0)	101 (59.1)
Stage II	23 (32.4)	27 (27.0)	50 (29.2)
Stage III	4 (5.6)	13 (13.0)	17 (9.9)
Stage IV	0	3 (3.0)	3 (1.8)
	**N = 72**	**N = 100**	**N = 172**
**Time to ART initiation in days, mean (SD), range**	**4.3 (8.0), 0.0–32.0**	**1.6 (5.1), 0.0–35.0**	**2.7 (6.6), 0.0–35.0**
**Time to ART initiation**			
0 days	41 (56.9)	81 (81.0)	122 (70.9)
1 day– 2 weeks	21 (29.2)	16 (16.0)	37 (21.5)
> 2 weeks—< 5 weeks	10 (13.9)	3 (3.0)	13 (7.6)
**Child ART regimen**	**N = 72**	**N = 100**	**N = 172**
ABC/3TC/ LPV/r	29 (40.3)	40 (40.0)	69 (40.1)
ABC/3TC/EFV	34 (47.2)	35 (35.0)	69 (40.1)
AZT/3TC/NVP	0	16 (16.0)	16 (9.3)
ABC/3TC/NVP	1 (1.4)	6 (6.0)	7 (4.1)
TDF/3TC/EFV	7 (9.7)	3 (3.0)	10 (5.8)
Unknown regimen	1 (1.4)	0	1 (0.6)
	**n = 72**	**n = 88**	**n = 160**
**Follow-up visits (median, IQR), range**	3.0 (2.0–3.0), 0.0–5.0	2.0 (1.0–2.0), 0.0–4.0	2.0 (1.0–3.0), 0.0–5.0
**Follow-up visits (median, IQR)**			
<2 years of age (n = 67, Kenya: 23, Uganda: 44)	3.0 (2.0–4.0)	2.0 (1.0–2.0)	2.0 (1.0–2.0)
2–9 years of age (n = 74, Kenya: 35, Uganda: 39)	3.0 (2.0–3.0)	2.0 (1.0–2.0)	2.0 (1.0–3.0)
> 9 years (n = 19, Kenya: 14, Uganda: 5)	2.5 (2.0–3.0)	2.0 (1.0–2.0)	2.0 (1.0–3.0)

*More than one response could be provided.

The majority of children started ART, including the two children with likely horizontal transmission; only two children in Kenya did not initiate treatment. The mean time from HIV diagnosis to ART initiation was 2.7 days (standard deviation: 6.6) with a maximum of 35.0 days (earlier in Uganda). Overall, 122 (70.9%) initiated ART on the same day as diagnosis, more frequently in Uganda than Kenya (81.0% versus 56.9%). The most common ART regimens were abacavir/lamivudine/lopinavir-ritonavir (69, 40.1%) and abacavir/lamivudine/efavirenz (69, 40.1%); 23 children (13.5%) received nevirapine combined with abacavir/lamivudine (n = 7) or zidovudine/lamivudine (n = 16) with most nevirapine-based regimens in Uganda. Ten children (5.8%) received tenofovir/lamivudine/efavirenz.

The median number of follow-up visits in the two-month period after diagnosis was 3.0 (IQR 2.0–3.0) and 2.0 (IQR 1.0–2.0) for children in Kenya and Uganda respectively. In total, 92.0% of children (Kenya: 72, Uganda: 88) had one or more follow-up visits and were not known to be deceased or defaulted by the two-month follow-up period (Fig 1); 10 of these children sought care and treatment services at a facility different from the testing facility. All five children who died were age ≤ two years; one child died in Kenya before follow-up data were collected, and all four children who died in Uganda were assessed as WHO Stage 3 and initiated ART prior to death. Nine children defaulted during follow-up (Kenya: 1, Uganda: 8).

## Discussion

There were multiple missed opportunities for prevention of HIV infection in newly diagnosed children living with HIV in Uganda and Kenya identified in our study, including gaps in ANC attendance, re-testing during pregnancy and breastfeeding, and initiation of maternal ARVs. Among study mothers and fathers of newly diagnosed children living with HIV in Uganda and Kenya in 2017–2018, 60 (45.5%) reported that the mother tested HIV-negative in ANC, suggesting that undetected incident HIV infection was a significant contributor to new infant infections. A significant proportion of HIV-positive mothers of newly diagnosed children did not receive ARVs during pregnancy or breastfeeding despite known HIV status. There were also gaps between maternal and child diagnoses, indicating possible delays in delivery of HIV testing services to HIV-exposed children. However, once HIV infection was diagnosed in children, there was accelerated linkage with HIV care and treatment services, with nearly all children initiated on ART within two weeks of diagnosis.

The proportion of women who attended ANC in both countries was lower compared to data from recent Demographic and Health Surveys. Eighty percent of study women in Kenya attended ANC compared to a national rate of 96% [20]. In Uganda, 86% of study women attended ANC versus a national rate of 97% [21]. In Kenya and Uganda, respectively, 38% and 35% of women delivered at home; the national rate of home deliveries in Uganda is considerably lower at 25% [21]. ANC serves as a key entry point for PMTCT services and both late pregnancy visits and the time of labor and delivery offer opportunities for retesting HIV-negative women [22,23].

Overall, most women attending ANC were offered HIV testing, though 11% of women in Uganda did not receive HIV testing. Among the 93 mothers who reportedly tested in ANC, nearly two-thirds tested HIV-negative. HIV retesting around the time of labor and delivery and during the postnatal period occurred more frequently in Uganda. A review of HIV service delivery in six sub-Saharan African countries, including Kenya and Uganda, revealed weaknesses with inconsistent implementation of provider-initiated HIV testing in ANC and less frequent retesting in pregnancy [24]. Barriers to retesting during pregnancy and postpartum persist at the individual, facility and health system levels [25]. Increasingly, incident infection is identified as a major contributor to new pediatric infections. The risk of HIV acquisition in pregnancy and early postpartum periods is elevated compared to non-pregnant women [26], and women with incident HIV infection had a greater risk of transmission of HIV to their infants compared to women with chronic HIV infection [27–29]. Implementing re-testing during pregnancy and the breastfeeding period and providing risk-reduction strategies, including pre-exposure prophylaxis to HIV-negative women at risk of HIV, are critical to the ability to achieve elimination of MTCT.

A considerable number of women with known HIV or who were newly diagnosed as HIV-positive in ANC were not initiated on ARVs during antenatal or postnatal care, largely due to ARVs not being offered by the health facility and disengagement from care. More women in Uganda were initiated on lifelong ART during breastfeeding than in Kenya; this may be due to a more established universal maternal ART (“Option B+”) program at the time of the majority of child diagnoses, which provided ART to all HIV-positive pregnant or breastfeeding women. Children in Uganda were younger (2.0 versus 3.6 years) and Option B+ was implemented earlier when compared to Kenya (2013 versus 2014) [30,31]. Given the critical importance of treatment and viral suppression in PMTCT and maternal health, it is not surprising that a disproportionate number of HIV-positive mothers of children newly diagnosed with HIV did not receive ARVs. Globally in 2017, 80% [61–>95%] of HIV-positive women received any ARV; in eastern and southern Africa, ARV access for pregnant women reached 93% [73–>95%] [1]. Women who do not receive ARVs are at high risk of transmitting HIV to their infants; barriers to ART initiation in the era of universal maternal ART include fear of lifelong treatment, feeling ill-prepared or too healthy to start treatment, and insufficient counseling [32,33]. Interventions shown to improve ART initiation and adherence include co-location of antenatal and ART services, strong linkages between services (e.g., peer escorts to treatment facilities), interventions to improve male partner involvement, electronic tracking systems, mHealth interventions, and differentiated care models to allow more intensive approaches to women newly diagnosed with HIV or those on ART with viral failure [24,34–36].

Nearly half of the children presented with one or more clinical symptoms, and more than half of the caregivers reported that their child becoming sick or weak was the impetus for testing the child. This aligns with previous research indicating higher yields of HIV-positive children at entry points where children tend to be sicker, such as IPD [7,37,38]. HIV-positive children should be identified before they are ill and initiated on treatment; however, the lack of clinical symptoms in a majority of children suggests continued gaps with case finding for older asymptomatic children.

In mothers diagnosed with HIV after delivery, 75% of their children were diagnosed with HIV within two months of maternal diagnosis, but one quarter of children were not diagnosed for several months to years following maternal diagnosis. Additionally, 39% of children of mothers with known HIV infection during pregnancy were not diagnosed until after age two years. Of those caregivers responding to why the child was not tested previously for HIV, 42.5% indicated that they were never offered testing for their children. Other studies have also documented institutional barriers to pediatric HIV testing, such as laboratory reagent stock-outs, weak tracking systems that do not follow up infants for testing and limited provider-initiated HIV testing for unknown or unreported child exposure at clinics outside of PMTCT/MCH [13,39].

Additionally, 14.4% of HIV-positive children had a prior test for HIV that was negative. A study in Lesotho found a 1.0% positivity rate (41/4051) among children of index clients 2–14 years of age who had been tested previously [11]. A facility-based study in Zimbabwe of 6–15 year olds found that children 12 years and older were less likely to be offered HIV testing [40]. This group also had the lowest testing acceptance rate, yet the median age of children testing HIV-positive was 11 years. Demand and supply side factors, such as low HIV risk perceptions and consenting issues (old enough to attend clinic alone but not old enough to consent to testing or to be offered testing by providers) may contribute to missed positive cases in this young adolescent age group [40,41]. More intensive, less traditional approaches to case finding may be required for perinatally infected but undiagnosed older children.

Community-based and index testing strategies accounted for a relatively small proportion of HIV-positive cases in our study. PITC accounted for the majority of cases primarily because children < 5 years comprised over 70% of our study population. When they presented to care in MCH/PMTCT or for under-five focused services, the children were tested because they were known to be HIV-exposed or because their mother just tested HIV-positive. Fewer children were identified through index testing procedures, which included active solicitation of contacts, tracing and testing. However, recent evidence suggests high positivity yields utilizing community index case testing. In Malawi, index testing in which contacts are offered community-based HIV testing, resulted in a 3.8% yield among children up to 15 years of age [9]. Other findings from Kenya provide evidence that utilizing adolescent siblings on ART and deceased clients with known HIV infection as the index clients and hybrid community/home-based testing were effective at identifying positive children 2–14 years of age [12,42].

Finally, our study shows that 99% of children initiated ART. Other studies have found 65%-95% of newly diagnosed children and adolescents were linked to HIV care and treatment [9,11,40]. However, despite all children being dispensed ART following diagnosis in Uganda, four children with advanced disease at diagnosis died. Additionally, nine (5.2%) children overall made no return visits for ART refills and other services during the study period. Community-based follow-up systems could be strengthened to ensure children newly initiated on ART, particularly those with advanced disease at time of ART start, and/or their caregivers, get the adherence and other support needed. Community worker programs in Zimbabwe and South Africa were associated with improved survival and virological outcomes among children newly enrolled on ART [43–45].

This study has some limitations. First, we did not assess transmission mode. We excluded two children who we could classify as likely horizontal transmissions from our review of mothers’ PMTCT cascade profiles. However, it is possible other children acquired HIV through a mode other than mother- to-child transmission. While MTCT accounts for most of the infections in children, there is still a need to assess other risk factors for HIV in this age group. Secondly, because study staff were not always able to accompany health workers for community-based testing, the total number of children diagnosed as HIV-positive in communities who did not present at the health facility for care and treatment during the study period is unknown. This exclusion could have resulted in an overestimation of the linkage to treatment rate. However, the number is expected to be small, based on programmatic data from the same period that shows high ART linkage rates from community testing in Kenya and high overall linkage rates in Uganda (where rates by testing modality cannot be determined from routine data). Thirdly, we are not able to draw conclusions regarding HIV-positive yield from different testing points from the cohort, as we only enrolled HIV-positive children. Moreover, expanding our study population to HIV-exposed but uninfected children and powering the study to compare differences in adherence to the PMTCT cascade among the two groups may have strengthened these findings or provided additional insights into the gaps contributing to child infection. However, the identification of gaps and sub-optimal fidelity to PMTCT service delivery are still important findings for program improvement to minimize the risk of transmission as much as possible. Risk factors, reflecting gaps identified in this study like lack of maternal ARV, are well documented in the literature [46–49].

## Conclusions

This study described multiple missed opportunities for prevention of HIV in newly diagnosed children living with HIV in Uganda and Kenya, including gaps in ANC attendance, re-testing during pregnancy and breastfeeding, and initiation of ARVs and possible delays in delivery of HIV testing services to HIV-exposed children. However, once HIV infection was diagnosed in children, nearly all children were promptly linked to treatment. Findings support improving PMTCT service delivery to HIV-negative women, including retesting during pregnancy and breastfeeding and emphasizing prevention messaging, strengthening referral and follow-up systems and other interventions to help ensure HIV-positive women receive ARVs in ANC and broader implementation of community-based approaches to HIV case finding to help ensure children not engaged in care receive HTS services.

## Supporting information

S1 Data (PDF)

S2 Data (PDF)
