# Supplementary material for: Active pediatric HIV case finding in Kenya and Uganda: A look at missed opportunities along the prevention of mother-to-child transmission of HIV (PMTCT) cascade
Source: PLoS One. 2020 Jun 2;15(6):e0233590. doi: 10.1371/journal.pone.0233590 (PMC7266341; doi:10.1371/journal.pone.0233590)
Supplement: S1 Data — (PDF) [file pone.0233590.s001.pdf]

Pediatric Active Case Finding of HIV through Facility and Community-based Testing Strategies (PedAC Study)

Interview date: ☐☐/ ☐☐/ ☐☐*(dd-mm-yy)* Interviewer ID: ☐☐

Health facility name: ______________________________ Health facility ID: ☐☐

Community catchment area (if applicable): ______________________________

Community testing organization (if applicable): ______________________________

Section A: Child HIV Testing Services

Questions in this section should be asked to health worker or abstracted from records.

1. Where was CHILD tested?
   - ☐ (1) Maternal and Child Health Clinic (MCH)/PMTCT
   - ☐ (2) Outpatient department (OPD)
   - ☐ (3) Inpatient Department (IPD)
   - ☐ (4) Home-based/door-to-door testing
   - ☐ (5) Malnutrition
   - ☐ (6) TB Clinic
   - ☐ (7) Under 5/EPI (Immunization) Clinic/Child Welfare Clinic (CWC)
   - ☐ (8) Voluntary Medical Male Circumcision (VMMC)
   - ☐ (9) Comprehensive Care Clinic (CCC)
   - ☐ (10) KYCS campaign
   - ☐ (11) OVC service organization
   - ☐ (12) Community outreach/child health days
   - ☐ (88) Other, specify______________________________

2. Through what approach was CHILD tested?
   - ☐ (1) PITC
   - ☐ (2) Family testing
   - ☐ (3) VCT
   - ☐ (88) Other, specify: ______________________________

Section B: Introductory Questions

Questions in this section should be asked to both biological and non-biological caregivers and emancipated minors (if applicable).

3. Who does CHILD live with? *(select all that apply)*
   - ☐ (1) Biological mother
   - ☐ (2) Biological father
   - ☐ (3) Sibling
   - ☐ (4) Step-parent
   - ☐ (5) Grandparent
   - ☐ (6) Other relative, e.g., aunt, uncle, cousin, etc.
   - ☐ (88) Other, specify: ______________________

4. Does CHILD meet the definition of emancipation per regulatory guidelines?
   - ☐ (1) Yes – *Skip to Q26*

☐ (2) No
☐ (77) N/A

5. What is the caregiver's relationship to the child?
   ☐ (1) Biological mother
   ☐ (2) Biological father
   ☐ (3) Sibling
   ☐ (4) Step-parent
   ☐ (5) Grandparent
   ☐ (6) Other relative, e.g., aunt, uncle, cousin, etc.
   ☐ (88) Other, specify: ______________________

Section C: Demographic Information

Questions in this section should be asked to both biological and non-biological caregivers.

6. What is the sex of the caregiver?
   ☐ (1) Male
   ☐ (2) Female

7. What is the caregiver's age? _____ _____ years

8. What is the caregiver's current marital status?
   ☐ (1) Married or living with partner as a couple
   ☐ (2) Never married or not living with partner
   ☐ (3) Divorced/Separated
   ☐ (4) Widowed

9. What is the highest level of education the caregiver completed at school?
   ☐ (1) Some Primary
   ☐ (2) All Primary
   ☐ (3) Secondary
   ☐ (3) Post-secondary/high school
   ☐ (4) Tertiary
   ☐ (5) Never attended school

Section D: Caregiver HIV Testing and ART History

All questions in this section should be asked to both biological and non-biological caregivers. Please remember you do not have to answer any questions if you are not comfortable.

10. What is the caregiver's HIV status?
    ☐ (1) HIV-positive
    ☐ (2) HIV-negative, tested in the last 3 months - *skip to Q16*
    ☐ (3) HIV-negative, tested more than 3 months ago - *skip to Q16*
    ☐ (4) Prefer not to answer - *skip to Q16*
    ☐ (99) Don't know (if it has been more than 3 months since the last HIV-negative test) - *skip to Q16*

11. Who has the caregiver told about his/her HIV-positive status? *(Select all that apply)*
    ☐ (1) No one
    ☐ (2) Partner
    ☐ (3) Another person

☐ (4) Just found out status today

12. Is the caregiver currently on ART?
    - ☐ (1) Yes – *skip to Q14*
    - ☐ (2) No

13. If not currently on ART, what are the reason(s)? *(select all that apply)*
    - ☐ (1) Stock-out of medication
    - ☐ (2) Experience of side effects
    - ☐ (3) Not permitted by spouse or other family member
    - ☐ (4) Did not want others to know I'm HIV-positive
    - ☐ (5) Took traditional medicine instead
    - ☐ (6) Experience of mental health issues
    - ☐ (7) Religious reasons
    - ☐ (8) Fear of lifelong treatment
    - ☐ (9) Financial reasons
    - ☐ (88) Other, specify: _________________

14. When was the caregiver first initiated on antiretroviral treatment? ☐☐/☐☐/☐☐ (*dd/mm/yy*) - *skip to Q16*
    - ☐ (99) Unknown - *skip to Q16*
    - ☐ (77) N/A-never initiated

15. If never initiated, why not? *(select all that apply)*
    - ☐ (1) Never offered by health facility
    - ☐ (2) Not eligible for treatment (high CD4 count)
    - ☐ (3) Not permitted by spouse or other family member
    - ☐ (4) Did not want others to know I'm HIV-positive
    - ☐ (5) Took traditional medicine instead
    - ☐ (6) Experience of mental health issues
    - ☐ (7) Religious reasons
    - ☐ (8) Fear of lifelong treatment
    - ☐ (9) Financial reasons
    - ☐ (88) Other, specify: _________________
    - ☐ (77) N/A

Caregivers other than the biological mother or father should skip to Section F (Q26).

Section E: MCH and HIV Health-seeking Behavior

Questions should be asked about the child diagnosed as HIV-positive (CHILD). The questions in this section should be directed towards the biological mother or father.

16. Did the mother attend antenatal care when pregnant with this CHILD?
    - ☐ (1) Yes
    - ☐ (2) No
    - ☐ (99) Don't know

17. Did the mother receive an HIV test while attending antenatal care with this CHILD?
    - ☐ (1) Yes
    - ☐ (2) No-known HIV-positive - *Skip to Q19*
    - ☐ (3) No-not offered - *Skip to Q19*
    - ☐ (4) No-offered, but refused - *Skip to Q19*

☐ (99) Don't know - *Skip to Q19*

18. What was the result of the mother's HIV test in ANC?
   ☐ (1) HIV-positive
   ☐ (2) HIV-negative
   ☐ (3) Prefer not to answer
   ☐ (99) Don't know

19. Did the mother undergo couples testing with CHILD's father as part of antenatal care?
   ☐ (1) Yes
   ☐ (2) No
   ☐ (99) Don't know

20. What was the result of the CHILD's father's HIV test in ANC?
   ☐ (1) HIV-positive
   ☐ (2) HIV-negative
   ☐ (3) Not tested
   ☐ (4) Prefer not to answer
   ☐ (99) Don't know

21. When did the mother learn about her HIV-positive status? ☐☐/☐☐/☐☐ (*dd/mm/yy*)
   ☐ (1) Not HIV-positive – *skip to Q26*
   ☐ (99) Don't know
   ☐ (77) N/A-never tested – *skip to Q26*

22. Did the mother receive antiretroviral medication during antenatal care with CHILD?
   ☐ (1) Yes – ARV prophylaxis– *skip to Q24*
   ☐ (2) Yes – ART prophylaxis to be taken during pregnancy/breastfeeding only– *skip to Q24*
   ☐ (3) Yes – ART to be taken for life – *skip to Q24*
   ☐ (4) No
   ☐ (5) Did not yet know HIV+ status – *skip to Q24*
   ☐ (99) Don't know– *skip to Q24*

23. If the mother did not receive antiretroviral medications during antenatal care with CHILD, why not?
   ☐ (1) Never offered by health facility
   ☐ (2) Did not yet know HIV status
   ☐ (3) Not permitted by spouse or other family member
   ☐ (4) Did not want others to know I'm HIV-positive
   ☐ (5) Took traditional medicine instead
   ☐ (6) Experience of mental health issues
   ☐ (7) Religious reasons
   ☐ (8) Fear of lifelong treatment
   ☐ (9) Financial reasons
   ☐ (88) Other, specify: ________________________________
   ☐ (99) Don't know

24. Did the mother receive antiretroviral medication during delivery or while breastfeeding CHILD?
   ☐ (1) Yes – ARV prophylaxis – *skip to Q26*
   ☐ (2) Yes – ART prophylaxis to be taken during breastfeeding only– *skip to Q26*
   ☐ (3) Yes – ART to be taken for life – *skip to Q26 (if answered 3 to Q22, this response should be the same)*
   ☐ (4) No

☐ (5) Did not yet know HIV+ status – *skip to Q26*
☐ (99) Don't know– *skip to Q26*

25. If the mother did not receive antiretroviral medications during delivery or while breastfeeding CHILD, why not?
☐ (1) Never offered by health facility
☐ (2) Did not yet know HIV status
☐ (3) Not permitted by spouse or other family member
☐ (4) Did not want others to know I'm HIV-positive
☐ (5) Took traditional medicine instead
☐ (6) Experience of mental health issues
☐ (7) Religious reasons
☐ (8) Fear of lifelong treatment
☐ (9) Financial reasons
☐ (88) Other, specify: ________________________________
☐ (99) Don't know

Section F: Child Medical, Growth and Developmental History

Questions in this section should be asked to both biological and non-biological caregivers and emancipated minors (if applicable).

26. What is the sex of CHILD?
☐ (1) Male
☐ (2) Female

27. What is CHILD's date of birth? ☐☐/☐☐/☐☐ *(dd-mm-yy)*
☐ (99) Don't know

28. Where was the CHILD delivered?
☐ (1) In a health facility
☐ (2) At home
☐ (88) Other, specify: ____________________________________
☐ (99) Don't know

29. How long was CHILD breastfed?
☐ (1) ___ months
☐ (77) N/A
☐ (99) Don't know

30. Was ARV prophylaxis (Nevirapine) given to CHILD?
☐ (1) Yes
☐ (2) No – *skip to Q32*
☐ (99) Don't know

31. If yes, how long was ARV prophylaxis (Nevirapine) given to CHILD?
☐ (1) Single dose only or duration of BF
☐ (2) For 6 weeks
☐ (3) For the duration of breastfeeding
☐ (88) Other, specify: ________________________________
☐ (99) Don't know

32. If ARV prophylaxis (Nevirapine) was not given to CHILD, why not? *(select all that apply)*
☐ (1) Never offered by health facility
☐ (2) Did not yet know HIV status/not HIV-positive

☐ (3) Not permitted by spouse or other family member
☐ (4) Did not want others to know I'm HIV-positive
☐ (5) Took traditional medicine instead
☐ (6) Experience of mental health issues
☐ (7) Religious reasons
☐ (8) Financial reasons
☐ (9) Delivered at home and did not return to health facility
☐ (88) Other, specify: ________________________________
☐ (99) Don't know

33. Was CHILD tested previously for HIV?
☐ (1) Yes
☐ (2) No - *skip to Q36*
☐ (99) Don't know - *skip to Q37*

34. At what age was the most recent HIV test (before today) performed? *(Select months if CHILD was under 24 months of age and enter the number of months. Select years if CHILD was older than 24 months and enter a whole number for the age.)*
☐ (1) ☐ ☐ months
☐ (2) ☐ ☐ years
☐ (99) Don't know

35. What was the result of the most recent HIV test (before today)?
☐ (1) HIV-negative
☐ (2) Indeterminate
☐ (3) Never received the result
☐ (99) Don't know

36. If CHILD had not been previously tested for HIV, why not? *(select all that apply)*
☐ (1) Never offered testing
☐ (2) Not permitted by spouse or other family member
☐ (3) Religious reasons
☐ (4) Caregiver feared child would experience stigma if positive
☐ (4) Caregiver did not feel s/he could manage CHILD's HIV care
☐ (88) Other, specify: ________________________________
☐ (99) Don't know

37. Why was CHILD tested today? *(select all that apply)*
☐ (1) First time testing had been offered
☐ (2) CHILD had become very sick/weak
☐ (3) Testing services offered in community (did not have to attend facility)
☐ (4) Just wanted to know HIV status of CHILD
☐ (5) Parent had become sick/died
☐ (6) Sibling had become sick/died
☐ (7) Parent was known to be/tested HIV-positive
☐ (8) Sibling was known to be/tested HIV-positive

☐ (88) Other, specify: ________________________________
☐ (99) Don't know

38. Was CHILD told about his or her status right after testing?
☐ (1) Yes
☐ (2) No
☐ (77) N/A (emancipated minor) – *skip to Q41*

39. Were other children in caregivers' care tested for HIV today?
    - ☐ (1) Yes
    - ☐ (2) No
    - ☐ (77) N/A (no other children in care)

40. If yes, who was tested?
    - ☐ (1) CHILD's siblings, if yes, how many? __________________
    - ☐ (2) Other children in family or living in household, if yes, how many? _____________
    - ☐ (3) Other adult family members, if yes, how many? ______________________

41. Who in CHILD's household has HIV? *(Select all that apply; do NOT include the respondent; do NOT anyone who tested HIV-positive today)*
    - ☐ (1) No one
    - ☐ (2) Caregiver's partner
    - ☐ (3) Caregiver's parent
    - ☐ (4) Other child (biological)
    - ☐ (5) Other child (non-biological)
    - ☐ (6) Other relative/friend living in home
    - ☐ (88) Other, specify: ______________________________

Interviews with emancipated minors should end here and participants thanked for their time.

Section G: Community and Facility-related Experiences

Questions in this section should be asked to both biological and non-biological caregivers.

42. Do you currently participate in any HIV support group at this or other facility?
    - ☐ (1) Yes
    - ☐ (2) No
    - ☐ (77) N/A-not HIV-positive

43. Do you belong to an ART buddy or community HIV support group?
    - ☐ (1) Yes
    - ☐ (2) No
    - ☐ (77) N/A-not HIV-positive

44. From whom do you seek HIV or other health-related information when you have questions? *(select all that apply)*
    - ☐ (1) No one
    - ☐ (2) Health care staff at this or another facility
    - ☐ (3) Community health worker
    - ☐ (4) HIV-positive peer
    - ☐ (5) Trusted friends or relatives
    - ☐ (88) Other, specify: _____________________

45. Do you think that the information or advice that you receive at the health facility or in the community helps you to take your HIV medicines correctly?
    - ☐ (1) Yes
    - ☐ (2) No
    - ☐ (77) N/A-not HIV-positive or not taking ART

Thank you for your time. We have completed the interview.
