# Supplementary material for: Active pediatric HIV case finding in Kenya and Uganda: A look at missed opportunities along the prevention of mother-to-child transmission of HIV (PMTCT) cascade
Source: PLoS One. 2020 Jun 2;15(6):e0233590. doi: 10.1371/journal.pone.0233590 (PMC7266341; doi:10.1371/journal.pone.0233590)
Supplement: S2 Data — (PDF) [file pone.0233590.s002.pdf]

Testing and Treatment Facilities/Communities	
**Health Facility Name where HIV testing was performed**	
**Health Facility ID**	
**Community Catchment Area** *(if applicable)*	
**Community Testing Provider** *(if applicable)*	
**Health Facility Name where ART services are being provided**	
**Health Facility ID** *(ID to be assigned later if non-study facility)*	

Select only one response unless otherwise indicated.

Child Follow-up Visits

Question	Response Code
1. Is the child receiving care at the facility where the child was referred to following diagnosis?	☐ (0) No –child receiving care at different facility<br>☐ (1) Yes<br>☐ (77) N/A –child not receiving care *(skip to Defaulter Tracing Section)*
2. Date of initial HIV antibody positive test:	__ __ /__ __ /__ __ *(dd/mm/yy)*<br>☐ (77) N/A for children <18 months (Uganda) or <12 months (Kenya)
3. Date positive DNA-PCR sample was collected:	__ __ /__ __ /__ __ *(dd/mm/yy)*<br>☐ (77) N/A<br>☐ (99) Date unknown
4. Date of positive DNA-PCR result receipt by mother/caregiver:	__ __ /__ __ /__ __ *(dd/mm/yy)*<br>☐ (77) N/A
5. Date of confirmatory HIV test:	__ __ /__ __ /__ __ *(dd/mm/yy)*<br>☐ (1) Confirmatory test not done<br>☐ (99) Date unknown
6. Result of confirmatory HIV test:	☐ (1) HIV-positive<br>☐ (2) HIV-negative<br>☐ (3) Indeterminate<br>☐ (4) Result never received
7. What illnesses/OIs did the child present with at time of HIV enrollment in care? *(select all that apply)*	☐ (1) None<br>☐ (2) Zoster<br>☐ (3) TB<br>☐ (4) Cryptococcal meningitis<br>☐ (5) Failure to thrive<br>☐ (6) Thrush

	☐ (7) Fever<br>☐ (8) Weight loss<br>☐ (9) Pneumonia<br>☐ (10) Chronic upper respiratory tract infections (cough)<br>☐ (11) Malnutrition<br>☐ (12) Persistent diarrhea<br>☐ (99) Unknown
8. What was the child's WHO stage at time of enrollment in care?	☐ (1) WHO Stage I<br>☐ (2) WHO Stage II<br>☐ (3) WHO Stage III<br>☐ (4) WHO Stage IV<br>☐ (99) Unknown
9. Date of ART initiation	__ __ /__ __ /__ __ *(dd/mm/yy)*<br>☐ (77) N/A (not started on ART)<br>☐ (99) Unknown
10. ART regimen	☐ (1) ABC/3TC/ LPV/r<br>☐ (2) ABC/3TC/EFV<br>☐ (3) ABC/3TC/NVP<br>☐ (4) ABC/3TC/AZT<br>☐ (5) AZT/3TC/ABC<br>☐ (6) TDF/3TC/EFV<br>☐ (7) TDF/3TC/DTG<br>☐ (77) N/A<br>☐ (88) Other, specify: ____________________
11. CD4 Count (if available)	☐ (1) __ __ __ cells/mm3<br>☐ (77) N/A
12. VL if available *(likely children under 18 months only)*	☐ (1) __ __ __ __ __ __ copies/ml<br>☐ (77) N/A

Date of visit *(dd/mm/yy)*	Services provided at this visit	Adherence assessment
13. __ __ /__ __ /__ __<br>☐ (77) N/A	☐ (1) ART pick-up<br>☐ (2) Pre-ART counseling<br>☐ (3) ART adherence counseling<br>☐ (4) OI Treatment<br>☐ (5) Nutrition assessment, counseling and support<br>☐ (6) TB screening/IPT<br>☐ (7) Non-HIV related medical visit/treatment	☐(1) Good<br>☐(2) Fair<br>☐(3) Poor<br>☐(4) Not done
14. __ __ /__ __ /__ __<br>☐ (77) N/A	☐ (1) ART pick-up<br>☐ (2) Pre-ART counseling<br>☐ (3) ART adherence counseling<br>☐ (4) OI Treatment<br>☐ (5) Nutrition assessment, counseling and support<br>☐ (6) TB screening/IPT<br>☐ (7) Non-HIV related medical visit/treatment	☐(1) Good<br>☐(2) Fair<br>☐(3) Poor<br>☐(4) Not done
15. __ __ /__ __ /__ __<br>☐ (77) N/A	☐ (1) ART pick-up<br>☐ (2) Pre-ART counseling<br>☐ (3) ART adherence counseling<br>☐ (4) OI Treatment<br>☐ (5) Nutrition assessment, counseling and support<br>☐ (6) TB screening/IPT<br>☐ (7) Non-HIV related medical visit/treatment	☐(1) Good<br>☐(2) Fair<br>☐(3) Poor<br>☐(4) Not done
16. __ __ /__ __ /__ __<br>☐ (77) N/A	☐ (1) ART pick-up<br>☐ (2) Pre-ART counseling<br>☐ (3) ART adherence counseling<br>☐ (4) OI Treatment<br>☐ (5) Nutrition assessment, counseling and support<br>☐ (6) TB screening/IPT<br>☐ (7) Non-HIV related medical visit/treatment	☐(1) Good<br>☐(2) Fair<br>☐(3) Poor<br>☐(4) Not done
17. __ __ /__ __ /__ __<br>☐ (77) N/A	☐ (1) ART pick-up<br>☐ (2) Pre-ART counseling<br>☐ (3) ART adherence counseling<br>☐ (4) OI Treatment<br>☐ (5) Nutrition assessment, counseling and support<br>☐ (6) TB screening/IPT<br>☐ (7) Non-HIV related medical visit/treatment	☐(1) Good<br>☐(2) Fair<br>☐(3) Poor<br>☐(4) Not done
18. __ __ /__ __ /__ __<br>☐ (77) N/A	☐ (1) ART pick-up<br>☐ (2) Pre-ART counseling<br>☐ (3) ART adherence counseling<br>☐ (4) OI Treatment<br>☐ (5) Nutrition assessment, counseling and support<br>☐ (6) TB screening/IPT<br>☐ (7) Non-HIV related medical visit/treatment	☐(1) Good<br>☐(2) Fair<br>☐(3) Poor<br>☐(4) Not done

Facility/Community Defaulter Tracing				
**Date of scheduled visit**	**Actions Taken**	**Date follow up attempts started** *(dd/mm/yy)*	**Able to contact client?**	**Tracing outcome**
19. __ __ /__ __ /__ __<br>☐ (77) N/A	☐(1) Phone call to client<br>☐(2) Phone call to treatment supporter<br>☐(3) Home visits<br>☐(4) Other, specify: ________________	__ __ /__ __ /__ __	☐(1) Yes<br>☐(2) No	☐(1) Rescheduled appointment<br>☐(2) Declined to attend<br>☐(3) Self-transfer to another HF<br>☐ (4) Change of residence<br>☐ (5) Too sick to attend health facility<br>☐ (6) Child deceased<br>☐(88) Other, specify: ________________
True,<br>20. __ __ /__ __ /__ __<br>☐ (77) N/A	☐(1) Phone call to client<br>☐(2) Phone call to treatment supporter<br>☐(3) Home visits<br>☐(4) Other, specify: ________________	__ __ /__ __ /__ __	☐(1) Yes<br>☐(2) No	☐(1) Rescheduled appointment<br>☐(2) Declined to attend<br>☐(3) Self-transfer to another HF<br>☐ (4) Change of residence<br>☐ (5) Too sick to attend health facility<br>☐ (6) Child deceased<br>☐(88) Other, specify: ________________
21. __ __ /__ __ /__ __<br>☐ (77) N/A	☐(1) Phone call to client<br>☐(2) Phone call to treatment supporter<br>☐(3) Home visits<br>☐(4) Other, specify: ________________	__ __ /__ __ /__ __	☐(1) Yes<br>☐(2) No	☐(1) Rescheduled appointment<br>☐(2) Declined to attend<br>☐(3) Self-transfer to another HF<br>☐ (4) Change of residence<br>☐ (5) Too sick to attend health facility<br>☐ (6) Child deceased<br>☐(88) Other, specify: ________________
22. __ __ /__ __ /__ __<br>☐ (77) N/A	☐(1) Phone call to client<br>☐(2) Phone call to treatment supporter<br>☐(3) Home visits<br>☐(4) Other, specify: ________________	__ __ /__ __ /__ __	☐(1) Yes<br>☐(2) No	☐(1) Rescheduled appointment<br>☐(2) Declined to attend<br>☐(3) Self-transfer to another HF<br>☐ (4) Change of residence<br>☐ (5) Too sick to attend health facility<br>☐ (6) Child deceased<br>☐(88) Other, specify: ________________

**Study-specific Tracing** ***(If child did not return within 6-8 weeks)***	
**Question**	**Response**
23. Since your child tested positive for HIV in [facility or community name], has your child been seen in any HIV care clinic?	☐ (0) No – *skip to Q28*<br>☐ (1) Yes, facility name? ________________________
24. Was your child retested for HIV at this other facility?	☐ (0) No, why not? ____________________________ – *skip to Q31*<br>☐ (1) Yes
25. What was the HIV test result?	☐ (1) HIV-positive<br>☐ (2) HIV-negative<br>☐ (3) Indeterminate<br>☐ (4) Result never received
26. What was the date your child was started on ART treatment?	__ __ /__ __ /__ __ *(dd/mm/yy)*<br>☐ (77) N/A - not started on ART, why not? ___________________________<br>☐ (99) Unknown
27. What ART regimen is the child taking? – *then skip to Q31*	☐ (1) ABC/3TC/ LPV/r<br>☐ (2) ABC/3TC/EFV<br>☐ (3) ABC/3TC/NVP<br>☐ (4) ABC/3TC/AZT<br>☐ (5) AZT/3TC/ABC<br>☐ (6) TDF/3TC/EFV<br>☐ (7) TDF/3TC/DTG<br>☐ (77) N/A<br>☐ (88) Other, specify: ____________________<br>☐ (99) Unknown
28. Why did you decide not to return to the clinic for your child's care? *(select all that apply)*	☐ (1) Financial reasons<br>☐ (2) Child does not have time (e.g., attending school)<br>☐ (3) Caregiver does not have time (e.g., work)<br>☐ (4) Child was enrolled in care at a different facility<br>☐ (5) Change of residence<br>☐ (6) Too sick to attend health facility<br>☐ (7) Child deceased<br>☐ (8) Mother/caregiver or other contact could not be reached<br>☐ (9) Child was well /didn't need treatment<br>☐ (10) Lack/fear of disclosure<br>☐ (88) Other, specify: __________________________
29. Provide further details on each of the responses selected.	

30. What would you need (or what would need to happen) for you to return to the health facility to have your child initiated on treatment? *(select all that apply)*	☐ (1) More income for food, transport, etc<br>☐ (2) Time to take off work<br>☐ (3) More assistance at home for household chores, caring for children, etc<br>☐ (4) Psychosocial support for caregiver<br>☐ (5) Psychosocial support for child<br>☐ (6) Assistance picking up drugs or taking child for services<br>☐ (7) Other, specify: ___________________________
31. Since the diagnosis of your child, have you been contacted by a community health worker or someone from the health facility?	☐ (0) No – *END HERE*<br>☐ (1) Yes, someone from the facility called me<br>☐ (2) Yes, someone from the facility visited me where I stay<br>☐ (3) Yes, someone from the facility called and visited me where I stay<br>☐ (4) Yes, a community health worker visited me where I stay
32. If yes, how many times were you contacted? (*indicate the number of times contacted for each mode on the lines provided, indicate 00 if no contact)*	☐ (1) By phone? __ __<br>☐ (2) By visit? __ __
33. During the call or visit, what did you discuss with the health/community health worker? *(select all that apply)*	☐ (1) Reasons why I have not attended the facility<br>☐ (2) How to address challenges faced with getting to clinic or getting care for the child<br>☐ (3) Consequences if child is not in care or on treatment<br>☐ (4) Child's health status<br>☐ (5) Health status of others in the household<br>☐ (6) A date to return to the facility<br>☐ (88) Other, specify: _________________________
34, Do you plan to take your child to the clinic to receive HIV care?	☐ (0) No<br>☐ (1) Maybe<br>☐ (2) Yes, soon<br>☐ (3) Yes, when he/she gets sick

Please provide any comments or explanation here:

Completed by: **Name:** **Name ID:**

Date Completed:
